# Investigating trends in antibiotic resistance of Escherichia coli isolated from clinical urine specimens in the Orkney Islands

**DOI:** 10.1099/mic.0.001514

**Published:** 2024-10-30

**Authors:** Lily Corse, Allison Cartwright

**Affiliations:** 1 Balfour Hospital, Foreland Road, Kirkwall, Orkney, Scotland; 2 Ulster University, Cromore Road, Coleraine, Northern Ireland

**Keywords:** antimicrobial resistance, *Escherichia coli*, surveillance, urinary tract infection

## Abstract

Urinary tract infections (UTIs) are extremely common, affecting people of all ages and health statuses. Although UTIs do not usually cause severe illness, in some cases they can lead to more serious complications, especially if their initial treatment is ineffective due to antimicrobial resistance (AMR). AMR is an increasing issue, exacerbated by misdiagnosis and inappropriate prescribing of antibiotics, thus facilitating further resistance. The aim of this study was to investigate the rates of AMR in *Escherichia coli* isolated from clinical urine specimens tested at the Balfour Hospital, Orkney, and determine trends related to patient risk factors. Antibiotic susceptibilities were tested for 100 isolates of uropathogenic *E. coli* using the VITEK 2 Compact (bioMérieux), and data were analysed using percentage resistance rates. Resistance rates were compared by patient sex, age and source (hospital versus community). The findings showed higher AMR in males compared with females, particularly for trimethoprim (TMP), with 52% in males and only 12% in females. AMR tended to be higher in *E. coli* isolated from hospital inpatients than from community specimens, except for amoxicillin (AMX) and co-amoxiclav. Finally, the study found that AMR of * E. coli* isolates was greater in patients aged over 50 than 18–50 years old, particularly for AMX and TMP. The highest resistance rates across all patient demographics were for AMX, implying that the use of this antibiotic for the treatment of *E. coli* UTIs is not appropriate.

**Impact Statement**

Antimicrobial resistance (AMR) is an ever-increasing issue globally, which is being exacerbated by the inappropriate use of antibiotics. Surveillance studies such as these are important in providing information on resistance profiles of pathogenic bacteria for geographical regions and providing a deeper understanding of trends in resistance to guide better, more informed prescribing practices, thus minimizing AMR.

## Data Summary

The authors confirm all supporting data, code and protocols have been provided within the article and through supplementary data files (available in the online version of this article).

## Introduction

Urinary tract infections (UTIs) are one of the most prevalent infections worldwide, accounting for an estimated 404.61 million cases globally in 2019 [1]. Around 95% of UTIs are caused by the colonization of bacteria in the urinary tract; however, other uropathogens include fungi such as *Candida* spp., viruses such as adenoviruses and parasites including *Schistosoma* and *Trichomonas* spp. [2]. Bacterial infections usually begin in the external regions of the genitalia and ascend the urethra. Here, bacteria may colonize the bladder, causing cystitis or infecting the kidneys [3]. Where the bacterial pathogen is able to colonize the kidneys, pyelonephritis can occur, and, in some cases, the infection may spread to the blood and cause urosepsis. Pyelonephritis and urosepsis are serious infections associated with high mortality – up to 40% for urosepsis [4] and 21% for emphysematous pyelonephritis [5]; therefore, UTIs must be treated promptly and effectively to prevent suffering and mortality. Immunocompromised patients and those with structural or functional abnormalities of the urinary system are at a greater risk of recurrent UTIs and are often susceptible to a wider range of uropathogens [6]. The most common bacterial cause of UTIs is *Escherichia coli*, causing up to 75% of uncomplicated UTIs and 65% of complicated UTIs [7] and is therefore the focus of this study.

The treatment of *E. coli* UTIs varies depending on clinical presentation, patient history and local resistance rates. The National Institute for Health and Care Excellence recommends empirically prescribing a short dose of first-line antibiotics such as nitrofurantoin (FT), trimethoprim (TMP) or amoxicillin (AMX) in the first instance for the treatment of uncomplicated UTIs [8]. Second-line antibiotics such as co-amoxiclav (AMC) should only be prescribed if first-line antibiotics are unsuitable for treatment due to allergies or resistance, as these are considered to be less effective than the first-line antibiotics and require a longer duration [9]. Third-line antibiotics used for the treatment of *E. coli* UTIs include fluoroquinolones such as ciprofloxacin (CIP). Although these antibiotics are often effective, they are associated with resistance and an increased risk of side effects.

Many *E. coli* strains and other members of the *Enterobacteriaceae* family are extended-spectrum β-lactamase (ESBL) producers, allowing them to actively destroy β-lactam antibiotics such as penicillins and cephalosporins. These resistant bacteria are capable of spreading naturally in the environment and causing severe and persistent infections, making them particularly dangerous in healthcare settings [10]. It is estimated that 60% of ESBL *E. coli* bacteraemia arises from UTIs [10]. ESBL UTI may be treated with co-amoxiclav, consisting of amoxicillin with the addition of clavulanic acid, which inhibits β-lactamase enzymes, allowing amoxicillin to bind and lyse bacterial cells effectively. Piperacillin–tazobactam (TZP) also works to inhibit β-lactamases and is useful for the treatment of ESBL infections if other antibiotics are not suitable [11].

Widespread overuse of broad-spectrum antibiotics has fast-tracked AMR [12]. Bacteria that possess resistance genes, such as * E. coli*, can transfer these genes to other bacterial cells by means of horizontal gene transfer [13]. When a sub-optimal dosage of an antibiotic is prescribed to treat a bacterial infection, natural selection allows cells possessing resistance genes to thrive, whilst other more susceptible cells are destroyed successfully, meaning that patients presenting with recurrent UTIs are potentially at a greater risk of resistant bacterial infections. Consequently, antibiotics that were once effective against the bacteria have themselves driven resistance. Resistance poses a major threat to the future of human health and the economy. In 2019, the World Health Organization estimated that if control of AMR is not reached, we can expect an estimated 10 million deaths caused by drug-resistant infections by 2050 [14]. In January 2019, the UK Government launched a 20-year plan to ‘contain and control’ AMR using a One Health approach [15]. Surveillance and research into patterns of AMR are key to fulfilling these ambitions.

Rapid and accurate identification and antibiotic susceptibility testing (AST) are essential for prescribing the appropriate antibiotics at the MIC and limiting AMR caused by ineffective treatment. Previous methods of AST were time-consuming and more complex, but with the introduction of automated AST systems such as the VITEK 2, multiple samples can be analysed simultaneously and results can be produced in as little as 18 h. The VITEK 2 system analyses standardized bacterial dilutions by recording colourimetric signals, fluorescence and turbidity in the reaction wells of the specially designed test cards to produce a report of bacterial identification down to genus level and an antibiotic susceptibility panel [16]. Automated systems such as these are essential in allowing clinicians to commence patients on the optimal antibiotic treatment in a timely manner and reducing the risk of treatment with an antibiotic to which the bacterium is resistant, therefore facilitating better treatment of UTIs. Surveillance of AMR is essential for infection control and improving antibiotic prescribing practices to minimize AMR. The purpose of this investigation was to determine the AMR profiles of *E. coli* isolated from clinical urine specimens of patients presenting with UTIs in the Orkney Islands and ascertain whether overall resistance rates were similar to the rest of Scotland.

## Methods

Analyses were conducted on 100 clinical urine specimens submitted to the Balfour Hospital microbiology laboratory, Orkney, Scotland, from 30 October to 29 November 2023, where the cause of UTIs was identified as *E. coli* following culture. *E. coli* resistance profiles were collected sequentially over this time period and as such, the data presented are for a randomly selected population.

### Microscopy and culture

Urine specimens were examined by direct microscopy, and 1 µl of each specimen was inoculated onto Chromogenic UTI Agar (E and O Labs Ltd.) using sterile disposable plastic loops and cultured for 18–24 h in air at 36 °C. Plates were examined for microbial growth, and specimens that yielded moderate (15–100 colonies) or heavy (>100 colonies) microbial growth were analysed on the VITEK 2 Compact. Colonies which were pink were presumptively identified as *E. coli* or were submitted to the VITEK 2 Compact for identification in addition to antimicrobial susceptibility testing.

### **Antibiotic** susceptibility testing

For each bacterial isolate, a few colonies were picked from the growth medium using a sterile cotton swab and inoculated into 3 ml of VITEK saline. The turbidity of the dilution was checked using a ‘Densichek’ (bioMérieux) until a 0.5 McFarland Standard was reached. One hundred and forty-five microlitres of this dilution was pipetted into a second 3 ml vial of VITEK saline, which was loaded into a processing rack with an AST-445 VITEK card for AST. If the bacterial species was unknown, the 0.5 McFarland dilution was also loaded into the processing rack with a VITEK identification card, allowing the identification and susceptibility testing to run concurrently. All isolates processed on the VITEK 2 Compact were inoculated onto Cystine Lactose Electrolyte Deficient (CLED) agar control plates, which were incubated overnight in the air. The control plates were assessed for bacterial growth prior to validating antibiotic susceptibilities to ensure that the bacterial isolate had been inoculated into the test dilution and that only the *E. coli* isolate was present. Isolates that flagged up on the VITEK 2 with unusual AMR patterns such as ESBLs were repeated on the VITEK 2 Compact if required and confirmed using the disc diffusion method with AmpC and ESBL detection set D68C impregnated discs (Mast Group) on Mueller–Hinton (MH) agar. The MH plates were incubated in the air at 36 °C for 18–24 h, and then the zones of inhibition were measured and a calculation Excel sheet (Mast Group) was used to interpret the results of the test.

Antibiotic sensitivities were recorded as ‘*R*’ denoting resistant, ‘*S*’ denoting sensitive and ‘*I*’ for ‘intermediate’ for 12 antibiotics routinely prescribed for the treatment of *E. coli* UTIs. These were as follows: gentamicin (GM), amoxicillin (AMX), co-amoxiclav (AMC), piperacillin-tazobactam (TZP), cephalexin (CN), cefotaxime (CTX), ceftazidime (CAZ), ciprofloxacin (CIP), meropenem (MEM), fosfomycin (FOS), nitrofurantoin (FT) and trimethoprim (TMP).

### Data collection and analysis

For each isolate of uropathogenic *E. coli* (UPEC), the patient’s sex, age, source (hospital inpatient or community) and antibiotic susceptibilities were recorded. Rates of resistance were calculated for each antibiotic in percentage using the formula (*R*/*N*) × 100, where *R* = number of resistant strains and *N* = total number of *E. coli* isolates tested. These resistance rates were compared for risk factors for UTI, including age, sex and patient location (hospital or community setting), and with data collected for * E. coli* isolated from urine specimens in Scotland in 2022 [17].

The European Association of Urology recommends the use of several first-line antibiotics included in this study for the treatment of uncomplicated UTIs, where the local resistance rates are less than 20% [18,19]. The threshold for a clinically significant rate of resistance was therefore defined as greater than 20% and significant difference as greater than 10% [20]. A two-tailed independent t-test was performed in Excel to determine if there was a significant difference at the 95% confidence level between the antibiotic resistance rates of *E. coli* isolates from urine for NHS Orkney in comparison with data for NHS Scotland. To compare antibiotic resistance by patient sex, source and age, independent t-tests were also conducted as above.

## Results and discussion

Antibiotic susceptibilities for 100 *E. coli* isolates from clinical urine specimens were analysed using the VITEK 2 Compact. The antibiotics included in the susceptibility panels were first-, second- and third-line antibiotics commonly used to treat *E. coli* UTIs and antibiotics used for the treatment of resistant strains of UPEC. The total number of resistant strains for each antibiotic is outlined in Table 1. Of the 100 *E. coli* isolates, 11 ESBL producers were identified by the VITEK 2.

**Table 1.** Antibiotic resistance rates of *E. coli* isolates from clinical urine specimens in the Orkney Islands

Antibiotic class	Antibiotic (VITEK 2 abbreviation)	*R*	*N*	%*R*
Aminoglycoside	GM	2	100	2
Penicillins	*AMX	44	100	**44**
	AMC	27	100	**27**
β-Lactamase inhibitor	TZP	1	98	1
Cephalosporins	CN	12	100	12
	CTX	11	98	11
	CAZ	8	100	8
Fluoroquinolone	CIP	8	100	8
Carbapenem	MEM	0	100	0
Phosphonic	FOS	3	100	3
Miscellaneous agents	FT	2	100	2
	TMP	21	100	**21**

*R,* number of resistant strains; *N,* total number of *E. coli* isolates tested and %*R,* antibiotic resistance rate (percentage resistance).

* Susceptibility results for AMX were inferred from ampicillin.

AMX showed a particularly high rate of resistance (44%), followed by AMC (27%) and TMP (21%), which are highlighted in bold in Table 1. All other antibiotics showed low levels of resistance in the 100 *E. coli* isolates. MEM was the only antibiotic to which there was no resistance in all 100 isolates. Very low resistance rates were found for TZP (1%), GM (2%), FT (2%) and FOS (3%).

### Comparison with NHS Scotland figures

**Fig. 1.**
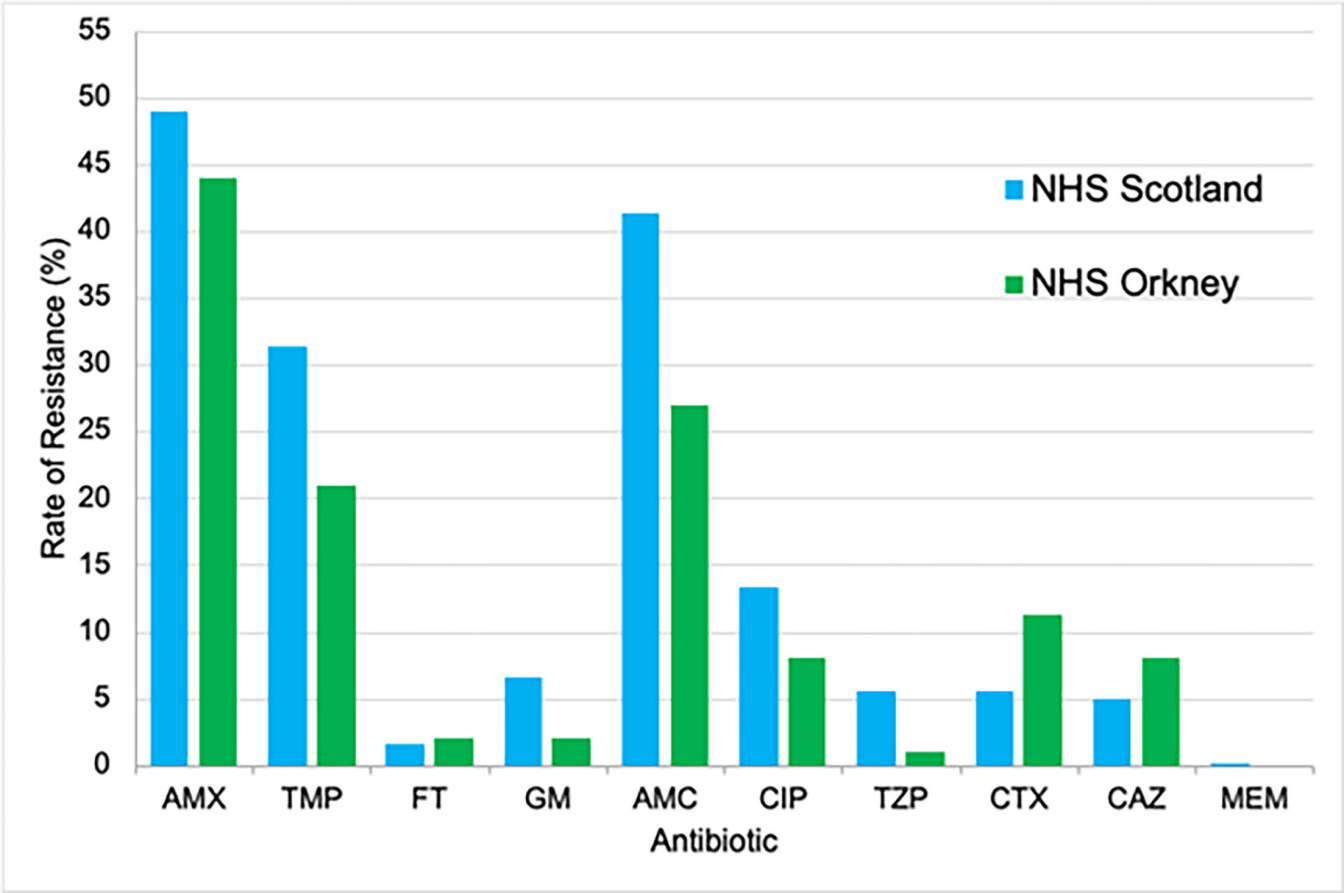
Comparison of antibiotic resistance rates *of E. coli* isolated from clinical urine specimens for NHS Scotland in 2022 [17] and NHS Orkney.

The resistance rates from this investigation at NHS Orkney were compared with data from NHS Scotland for a total of 142 615 * E. coli* isolates from urine specimens of patients presenting with UTIs in 2022 (Fig. 1). The highest rate of resistance in NHS Orkney was to AMX (44%), which was similar to the national rates (49%). AMC produced the second-greatest resistance rate in NHS Orkney at 27%, which was significantly lower than NHS Scotland (41%), showing that Orkney had a lower-than-expected issue with resistance to this antibiotic. TMP produced the third highest rate of resistance in both NHS Orkney and NHS Scotland, 21 and 31%, respectively, which was significantly lower for NHS Orkney than the national rate. The investigation showed that UPEC had the lowest rate of resistance to the carbapenem, MEM (0%), which was the same as NHS Scotland. This result also correlates with a study by Critchley *et al*. [21], who found that all *E. coli* strains from urine for 18 European countries in 2018 were susceptible to MEM. Carbapenems such as MEM and ertapenem are potent antibiotics against *E. coli* but are more expensive, administered intravenously and are not routinely prescribed for the treatment of UTIs [22], which may partially explain their low resistance rate for *E. coli*. All other antibiotics had similar resistance rates to that of NHS Scotland and, in general, were lower than the national figures, however not to a significant level (*t* = 1.734; *df* = 18; *P* = 0.635). Only CTX and CAZ resistance rates were slightly higher for NHS Orkney than expected (11 and 8%, respectively); however, these were not significantly different to NHS Scotland (5% for both antibiotics).

### Comparison by patient sex

**Fig. 2.**
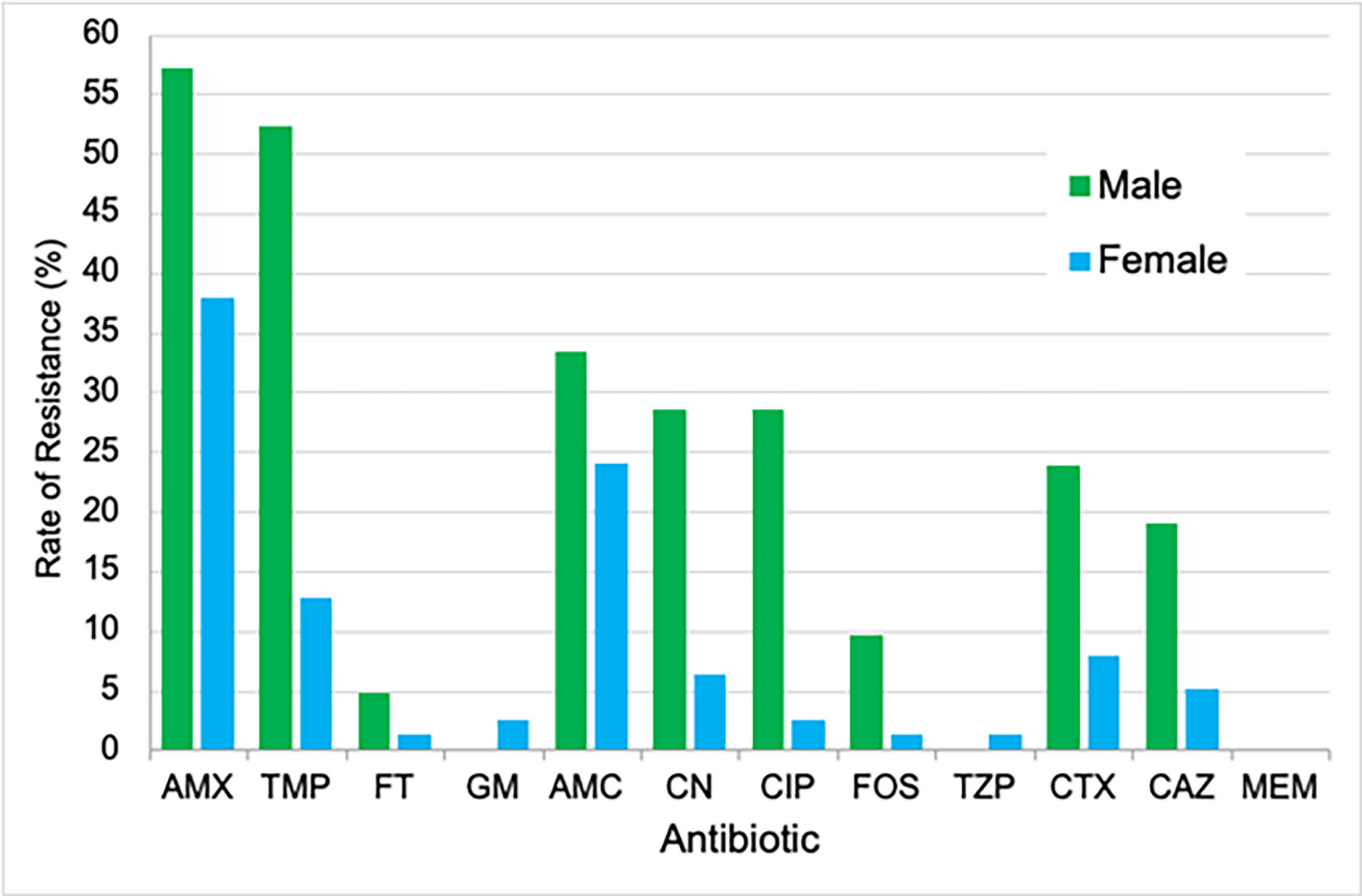
Antibiotic resistance rates of *E. coli* isolated from clinical urine specimens analysed by patient sex.

Of the total 100 *E. coli* urine specimens collected, there were a total of 21 from males and 79 from females. UTIs are more common in females due to the anatomy and physiology of the female urogenital system [20,23], which supports the initial findings of this investigation. When the overall resistance profiles were compared, there was no significant difference between sexes (*P* = 0.06). However, the t-test result was borderline, possibly owing to some antibiotics having very low resistance in both sexes. The antibiotics with the highest resistance rates in both males and females were AMX, TMP and AMC (Fig. 2). The resistance rates for several of the 12 antibiotics showed a difference of greater than 10% between males and females, making them significantly different. A resistance rate of 52% was recorded for TMP in males, which was 40% higher than in females. The second greatest difference in %*R* was observed for CIP, with 29% in males and only 3% in females, giving a difference of 26%. The resistance rates for CN, AMX, CTX and CAZ were also significantly higher in males. This correlates with the findings from previous studies on the association of patient sex with antimicrobial-resistant *E. coli* UTIs [19,20]. FT, GM, FOS, TZP and MEM had sufficiently low resistance rates (<20%) in males. These results suggest that the use of FT in the community and GM in hospital settings as first-line antibiotics is the most suitable treatment option for uncomplicated *E. coli* UTIs in males, and TMP, CIP, penicillins and cephalosporins are inappropriate for use. A study by Lagacé-Wiens *et al*. [24] suggests that the use of fluoroquinolones and TMP for the treatment of UTIs in males is inappropriate and supports the findings of this investigation that patient sex should be taken into consideration when prescribing antimicrobial therapies for UTIs. Whilst UTIs are more common in females, males are more susceptible to complicated UTIs associated with prolonged antibiotic treatment, which is a significant factor in driving AMR.

The National Institute for Health and Care Excellence recommends prescribing FT or TMP as a first-line antibiotic to treat uncomplicated UTIs in non-pregnant women [8]. The findings of this investigation reflect these recommendations, as both of these antibiotics showed a low rate of resistance (TMP 13% and FT 1%). The only two antibiotics that showed a significant rate of resistance in females were AMX (38%) and AMC (24%), suggesting that these antibiotics should be reviewed further to determine whether they are appropriate for the treatment of *E. coli* UTIs in females.

### Comparison of hospital and community sources

**Fig. 3.**
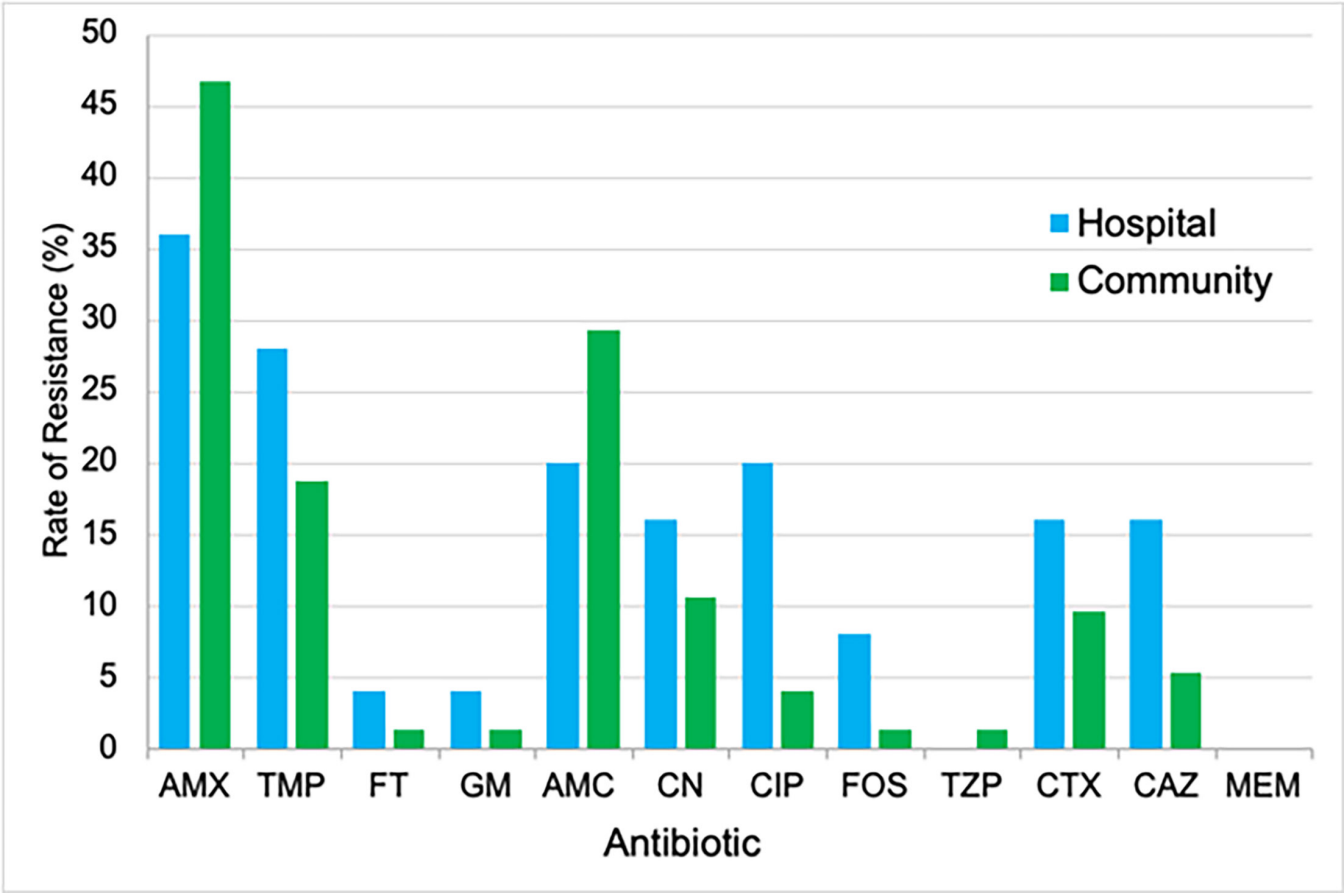
Antibiotic resistance rates of *E. coli* isolated from clinical urine specimens categorized by hospital and community sources.

*E. coli* accounts for ~90% of community-acquired and 50% of hospital-acquired UTIs [25]. The total number of hospital inpatient urine specimens acquired for this investigation was 25, and there were 75 community specimens. The results showed that overall resistance was higher in hospital specimens compared with community specimens for 8 of the 12 antibiotics (Fig. 3), but the overall resistance profiles were not significantly different (*P* = 0.549). In hospital patients, resistance was only clinically significant for AMX and TMP (36 and 28%, respectively). In community specimens, %*R* was particularly high for AMX (47%) and was significantly higher than hospital specimens, which was unexpected. Resistance to AMC was clinically significant in community specimens (29%) but not clinically significant in hospital specimens (9%). The resistance rate in hospital specimens to CIP was 20%, which was of clinical significance. This was 16% higher than the community specimens, meaning that CIP had the greatest difference between the two groups. This correlates with the results of a study by Fasugba *et al*. [26], who found that CIP resistance was significantly higher in hospital inpatients than in the community setting. The higher rate of AMR in hospital specimens is unsurprising, given that many inpatients are likely to have a previous history of UTIs, prior use of antibiotics, suffer from other comorbidities or immunosuppression and may have been catheterized [27]. However, previous studies state that hospital-acquired UTI specimens should only be included for comparison to community specimens if they are collected at least 48 h after admission so that it can be presumed that the infection began within the hospital setting and not prior to patient admission [28,29]. This method was not applied to this investigation due to a lack of clinical data; therefore, some ‘hospital’ specimens may have been included for patients who became infected in the community and did not acquire infection in the hospital setting.

### Comparison by patient age

**Fig. 4.**
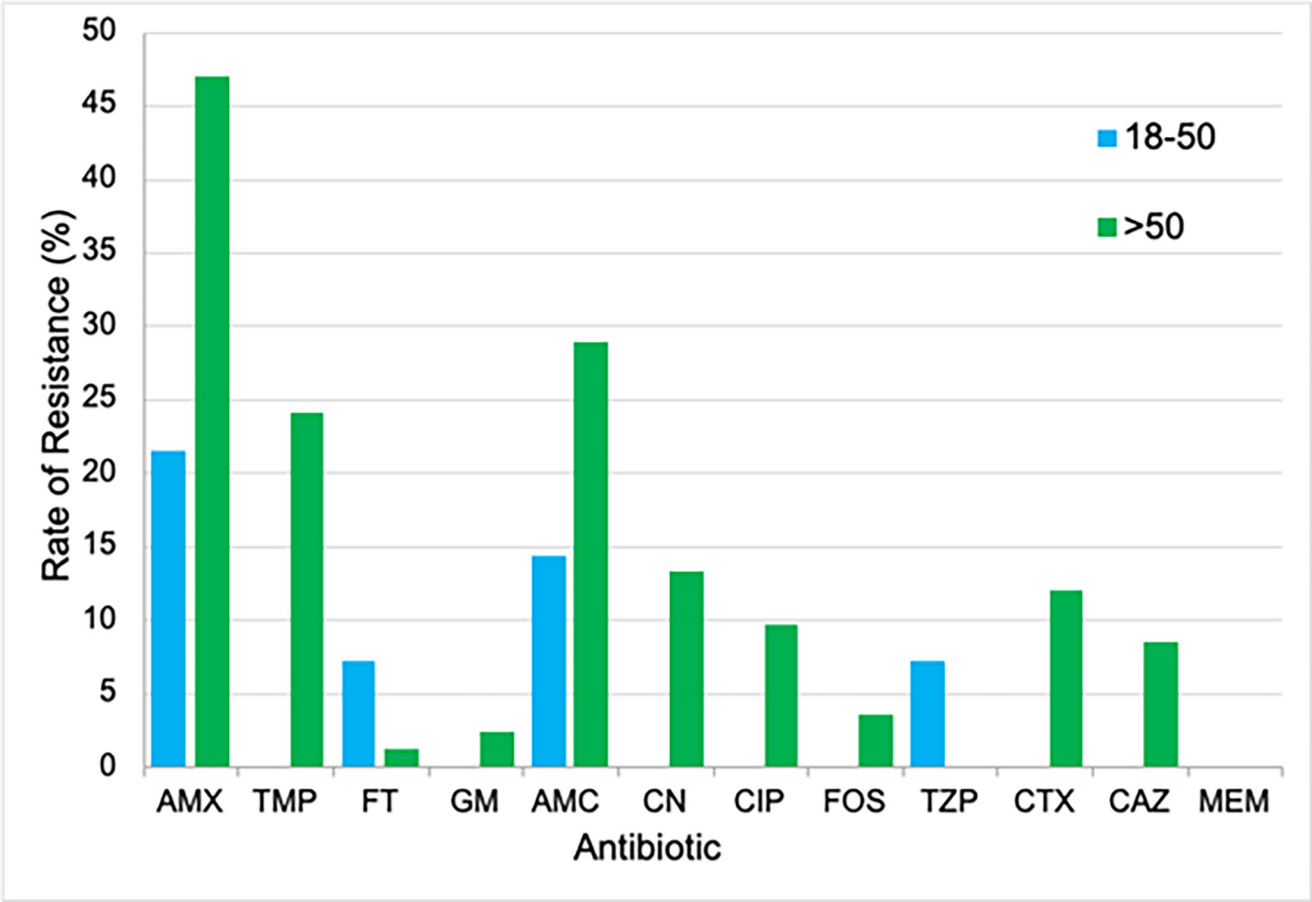
Antibiotic resistance rates of *E. coli* isolated from clinical urine specimens analysed by patient age group.

The specimens acquired for this investigation were collected from patients with an age range of 5−97 years with a mean age of 68 years. For the purpose of investigating a possible correlation between age and antibiotic resistance, the data were grouped under 18, 18−50 and over 50 years of age. There were only three patients in the under-18 age category, so this was excluded from the analysis. However, there were 14 patients aged 18–50 and 83 patients aged >50 years. When compared using a t-test, the difference in antibiotic profile between age groups was not significant (*P* = 0.087). Both the 18–50 and >50 age groups had clinically significant rates of resistance to AMX (21 and 47%, respectively; Fig. 4). The 18–50 year group showed a low level of resistance to all antibiotics except AMX (21%). Only FT and TZP had a higher %*R* for 18–50 years old than other age groups, but both were only 7%. In addition to AMX, the >50 years group also yielded a clinically significant rate of resistance to AMC (29%) and TMP (24%), which reflected the overall results for the 100 samples. A study by Huang *et al*. [30] found that the highest rates of antibiotic resistance were for ampicillin, TMP and cephalosporins for children aged 14 years and under, which is presumed to be a result of inappropriate prescription of antibiotics, but due to the very low sample size for under 18 years old, the data collected in this investigation could not be compared meaningfully. Several studies have found that AMR increases with age [31,32]. Multiple factors contribute to this relationship, including reduced immunity, increased risk of underlying health conditions, use of indwelling catheters and increased or repetitive use of antibiotics. In order to confirm a correlation between increasing age and increasing rate of AMR in *E. coli* UTIs, a larger, more evenly spread sample of the population would be required.

### ESBL producers

The most frequently prescribed antibiotics worldwide are β-lactams [33]. This group includes antibiotics such as AMX and cephalosporins, which are commonly prescribed for the treatment of UPEC and have been in use for many years. Some species of bacteria, including *E. coli*, possess transposable genes encoding the production of β-lactamase enzymes which hydrolyse β-lactam antibiotics. Overuse of these antibiotics has driven the resistance, and ESBL producers are becoming more widespread [34]. Suspected ESBLs isolated from patients who had a previous history of ESBL *E. coli* in their urine resulted in ESBLs without further confirmation. Those isolated from patients who had no known history of ESBL infection were confirmed using a disc diffusion detection test (Mast Group). This detection kit consisted of four discs impregnated with cefpodoxime, cefpodoxime and ESBL inhibitor, cefpodoxime and AmpC inhibitor and cefpodoxime with inhibitors of both AmpC and ESBL. A cross-sectional study of urine samples from 1013 laboratories in France in 2021 [35] found that the mean prevalence of ESBL-producing *E. coli* from urine was 3%. This contrasted with the 11% of *E. coli* isolates in the present study found to be ESBL producers, although a difference of 8% was not clinically significant. Five ESBLs were isolated from patients with a previous history of ESBL UTIs, which is a risk factor for recurrence. The distribution of male/female and hospital/community ESBL producers was roughly equal (6/5 and 4/7, respectively). This was unexpected as male sex and hospitalization are both risk factors for ESBL UTIs [36]; however, a larger population size may have yielded more apparent trends and 11 samples were not great enough to produce reliable results. Ten of the 11 ESBL-producing *E. coli* isolates were collected from patients over the age of 50 years, which correlates with the findings of several studies showing that ESBL UTIs are associated with increased age [37]. The remaining ESBL was isolated from the urine specimen of an 8-year-old child.

Treatment options are limited for resistant infections such as ESBL UTIs, as a high proportion of antibiotics commonly prescribed for the treatment of UPEC are β-lactams. ESBLs can be inhibited by clavulanic acid and tazobactam, but AmpC β-lactamase producers are resistant to these antibiotics and can hydrolyse several other antibiotics, including cephalosporins, aminopenicillins, cefoxitin and monobactam [11]. Due to the high level of resistance associated with these infections, parenteral administration of carbapenems may be required [38]. However, some *E. coli* strains are also able to produce carbapenemases, which break down carbapenem antibiotics. These are known as carbapenemase-producing *Enterobacterales* (CPE) and are even more resistant to antimicrobials than ESBLs and AmpC producers. CPE treatment options generally include second-line antibiotics such as aminoglycosides and FOS, and combination therapy tends to be more effective for patients with high-risk conditions [39]. There were no AmpC producers or CPEs identified in this investigation. The prevalence of CPE is currently low; a UK-based study by Trepanier *et al.* [40] found that of the 102 urine samples collected, 0.02% were CPE producers, which correlates with the findings of this study.

### Limitations

Although the results of this investigation compared well with similar studies, and the independent t-test proved that there was no statistically significant difference between resistance rates of UPEC for NHS Orkney and NHS Scotland, there were some limitations identified. The overall sample size of 100 *E. coli* isolates may have been too small to accurately reflect resistance rates for the wider population of the Orkney Islands, which is home to ~22 000 people. Additionally, there were only three urine *E. coli* isolates for patients under the age of 18 years, which was not sufficient to fully investigate a trend in age-related AMR, and these results were therefore discounted from analyses. If this investigation was to be repeated, it would add value to include a larger sample size that may reflect a true population more accurately. However, this study does provide a resistance profile baseline for *E. coli* UTI in the Orkney Islands and is comparable with the rest of the UK as the NHS follows similar antimicrobial prescribing guidelines.

## Conclusions

*E. coli* isolated from urine specimens in the Orkney Islands exhibited a clinically significant level of resistance to AMX, AMC and TMP. There was a lower level of resistance to AMC and TMP compared with figures published for NHS Scotland for 2022, but the overall resistance rates were similar to national data and several other studies. There was a correlation between male sex and increased antibiotic resistance, which was expected as males are prone to more complicated UTIs associated with prolonged antibiotic use. This trend highlights the importance of taking patient sex into consideration when prescribing antibiotic treatment for *E. coli* UTI. The results showed that hospital-associated *E. coli* UTIs were more resistant than community-acquired UTIs. AMR rates were higher overall for hospital specimens except for AMX and AMC, which were the only two antibiotics with clinically significant resistance rates for community specimens. It was seen that the greater antibiotic resistance was related to increased patient age when comparing 18–50 with >50 years old. Resistance was significantly higher for AMX, AMC, TMP, CN and CTX for patients over the age of 50 years compared with those aged 18–50 years. Across all patient demographics, *E. coli* urine isolates produced very high resistance rates to AMX, which implied that this antibiotic is not suitable for the treatment of UPEC. There were no AmpC or CPE isolates identified in this investigation; however, there were 11 ESBL isolates (11%), which was higher than previous studies but not to a clinically significant level. Surveillance of AMR is essential for guiding prescribing practices and informing on trends and risk factors for resistance. Continual studies such as this are required to control and prevent further AMR as well as monitoring the effectiveness of current intervention measures so that the UK may reach its goal to contain and control AMR.

## supplementary material

10.1099/mic.0.001514 Uncited Supplementary Material 1.

## Abbreviations

AMC: co-amoxiclav
AMR: antimicrobial resistance
AMX: amoxicillin
AST: antibiotic susceptibility testing
CAZ: ceftazidime
CIP: ciprofloxacin
CLED: Cystine Lactose Electrolye Deficient agar
CN: cephalexin
CPE: carbapenemase producing *Enterobacterales*
CTX: cefotaxime
ESBL: extended-spectrum β-lactamase
FOS: fosfomycin
FT: nitrofurantoin
GM: gentamicin
MEM: meropenem
MH: Mueller-Hinton
NHS: National Health Service
TMP: trimethoprim
TZP: piperacillin–tazobactam
UPEC: uropathogenic *Escherichia coli*
UTI: urinary tract infections
